# Effects of spray dried plasma on litter growth performance and oxidative stress and inflammation of sows kept in a hot environment

**DOI:** 10.1186/s40104-024-01139-9

**Published:** 2025-01-24

**Authors:** Hannah M. Bailey, Natalia S. Fanelli, Joy M. Campbell, Hans H. Stein

**Affiliations:** 1 Department of Animal Sciences, University of Illinois, Urbana, IL 61801 USA; 2 Present Address: Nestlé Purina PetCare Company, St. Louis, MO 63102 USA; 3 APC LLC, Ankeny, IA USA

**Keywords:** Cytokines, Lactation, Sows, Spray dried plasma, Weanling pigs

## Abstract

**Background:**

Feeding spray dried plasma (SDP) to weanling pigs improves growth, but there is a lack of research on how SDP impacts oxidative stress and inflammatory response in lactating sows, and performance of their piglets after weaning. Therefore, an experiment was conducted to test the hypothesis that sows fed a diet with SDP in late gestation and lactation have improved reproductive performance and reduced inflammation compared with sows fed no SDP. The second hypothesis was that pigs weaned from sows fed 0.5% SDP in lactation have reduced diarrhea incidence and improved growth performance during the initial 14 d of the nursery period regardless of dietary SDP.

**Results:**

The percent of low vitality or starved pigs during lactation was less (*P* < 0.05) from sows fed 0.5% dietary SDP compared with sows fed the diet without SDP. Dietary SDP did not influence oxidative stress markers in the plasma of sows, but serum cytokines increased (*P* < 0.05) in sows fed the diet with 0.5% SDP compared with sows fed the diet without SDP. Pigs weaned from young sows fed no SDP or from mature sows fed 0 or 0.5% SDP had a greater gain to feed ratio when fed a phase 1 diet containing 6% SDP compared with pigs fed a diet without SDP, but the gain to feed ratio of pigs weaned from young sows fed 0.5% dietary SDP was not affected by dietary SDP in phase 1 (interaction, *P* < 0.05). Regardless of sow treatment, pigs fed a phase 1 diet with 6% SDP had greater (*P* < 0.05) growth performance than pigs fed a phase 1 diet without SDP, and pigs fed the phase 1 diet with 6% SDP had reduced (*P* < 0.05) diarrhea incidence in phase 1.

**Conclusions:**

Feeding 0.5% dietary SDP to sows may reduce the number of mummified pigs and increase pig vitality during lactation, but adding 0.5% SDP to sow diets during lactation did not improve post-weaning performance of pigs fed a starter diet with 6% SDP.

## Background

Sows kept in environments with high temperatures are under greater stress compared with sows located in environments with lower average temperatures [1]. Increased stress during gestation can result in reduced feed intake, and therefore, the sow may enter a negative energy balance before farrowing [1, 2]. This can lead to an increased number of still born pigs, reduced litter size and pig birth weight, and increase in the inflammatory response of the sow during farrowing [1]. In addition, increased heat stress during early lactation may result in increased oxidative stress [3], reduced litter weight gain, reduced pre-weaning survival, and reduced weaning weight of pigs [4, 5].

Sows in a high stress environment had greater feed intake and a more efficient immune response if spray dried plasma (SDP) was included in the diet due to the presence of bioactive compounds such as immunoglobulins, peptides, and cytokines in SDP, which are believed to enhance immune function in sows and their piglets [6]. In addition, inclusion of 0.5% or 2.5% SDP in diets fed to gestating sows 4 d before parturition reduced the number of still born pigs [6]. Lactating sows fed a diet with 0.5% SDP had increased feed intake and increased weaning weight of pigs [5]. However, data for effects of SDP on sow oxidative stress and inflammatory response, especially cytokine synthesis, and subsequent performance of the offspring have not been reported. Feeding weanling pigs a diet with up to 6% SDP increases average daily gain and feed intake during the initial 2 weeks post-weaning [7]. However, research to determine if growth performance of weanling pigs can be further improved if they are weaned from sows fed a diet containing SDP is limited.

The current experiment was conducted to determine effects of SDP supplementation during sow gestation and lactation, as well as litter performance, oxidative stress, and inflammation in sows housed in a hot environment and subsequent litter performance 14 d post-weaning. The following hypotheses were tested: 1) addition of 0.5% SDP to diets fed to sows in late gestation and lactation reduces the number of stillborn pigs, increases litter weight, and reduces biomarkers of oxidative stress throughout lactation; and 2) pigs weaned from sows fed 0.5% SDP have reduced diarrhea incidence and improved growth performance during the initial 14 d of the nursery period.

## Methods

The Institutional Animal Care and Use Committee at the University of Illinois reviewed and approved the protocol for the experiment before animal work was initiated.

### Animals, diets, and experimental design

A total of 79 Camborough sows were bred to Line 800 boars (PIC, Hendersonville, TN, USA), and on d 107 of gestation, sows were moved to the lactation facility. Sows were randomly assigned to dietary treatments in a randomized complete block design with date of breeding used as the blocking factor. There were 2 dietary treatments (i.e., 0 or 0.5% SDP) and 2 parity groups (young, parity 1 and 2; and mature, parity ≥ 3); therefore, there were a total of 39 and 40 replicate sows receiving each of the 2 dietary treatments, and between 17 and 23 replicate sows per diet within parity group. The nursery phase of the experiment, where weaned pigs remained with their litter mates, used a split-plot design with sow treatment as the main plot and nursery diet (a phase 1 diet with 0 or 6% SDP) as the sub-plot. Wean group was used as the blocking factor for the nursery experiment, and there were 8 treatment groups in the nursery with 15 to 19 replicate pens of 4 or 5 pigs per pen for each of the 4 sow treatment groups.

Spray dried plasma (Appetein B) was sourced from APC Inc., Ankeny, IA, USA, and the same batch was used in the sow and post-weaning diets. Five diets were prepared (Tables 1 and 2); 2 lactation diets, without or with 0.5% SDP, were fed to sows from d 107 of gestation and until weaning. Two phase 1 diets, without or with 6% SDP, were fed to pigs for 14 d after weaning, and one common phase 2 diet without SDP was fed to all pigs for an additional 22 d. Vitamins and minerals were included in all diets to meet or exceed current nutritional requirement estimates of sows or nursery pigs [8]. A sample of the main ingredients and of all diets were collected at the time of diet mixing and these samples were used for chemical analysis.

**Table 1:** Ingredient composition of experimental diets (as-fed basis)

Item, %	Lactation period			Nursery period		
	Basal		Creep feed	Phase 1		Phase 2
Spray dried plasma, %:	‒	0.50	4.75	‒	6.00	‒
Spray dried plasma	‒	0.50	4.75	‒	6.00	‒
Corn (ground)	67.41	66.97	41.97	40.65	42.81	48.30
Soybean meal (45% crude protein)	25.00	25.00	22.00	25.00	25.00	25.00
Soybean hulls	10.00	10.00	‒	‒	‒	‒
Whey powder (dried)	‒	‒	25.00	20.00	20.00	15.00
Soy protein concentrate	‒	‒	‒	8.00	‒	5.00
Soybean oil	4.00	4.00	‒	3.10	3.10	3.50
Choice white grease	‒	‒	2.00			
Limestone (ground)	0.78	0.81	1.35	0.95	1.20	0.99
Dicalcium phosphate	1.69	1.65	0.45	1.10	0.80	1.00
Sodium chloride	0.40	0.40	0.20	0.10	0.10	0.10
Choline	0.10	0.10	‒	‒	‒	‒
L-Lys HCl	0.11	0.07	0.35	0.38	0.29	0.36
DL-Met	‒	‒	0.15	0.12	0.15	0.16
L-Thr	0.01	‒	0.08	0.10	0.05	0.09
Zinc oxide	‒	‒	0.40	‒	‒	‒
Vitamin mineral premix^a^	0.50	0.50	‒	0.50	0.50	0.50
Vitamin premix^b^	‒	‒	0.20	‒	‒	‒
Trace mineral premix^c^	‒	‒	0.35	‒	‒	‒
Pulmotil 18^d^	‒	‒	0.75	‒	‒	‒

^a^The vitamin-micromineral premix provided the following quantities of vitamins and micro minerals per kg of complete diet: vitamin A as retinyl acetate, 11,136 mg; vitamin D_3_ as cholecalciferol, 2,208 mg; vitamin E as DL-alpha tocopheryl acetate, 66 mg; vitamin K as menadione dimethylprimidinol bisulfite, 1.42 mg; thiamin as thiamine mononitrate, 0.24 mg; riboflavin, 6.59 mg; pyridoxine as pyridoxine hydrochloride, 0.24 mg; vitamin B_12_, 0.03 mg; D-pantothenic acid as D-calcium pantothenate, 23.5 mg; niacin, 44.1 mg; folic acid, 1.59 mg; biotin, 0.44 mg; Cu, 20.0 mg as copper sulfate and copper chloride; Fe, 126.0 mg as ferrous sulfate; I, 1.26 mg as ethylenediamine dihydriodide; Mn, 60.2 mg as manganese sulfate; Se, 0.30 mg as sodium selenite and selenium yeast; and Zn, 125.1 mg as zinc sulfate

^b^The vitamin premix provided the following quantities of vitamins per kg of complete diet: vitamin A, 681,818 mg; vitamin D_3_, 68,181 mg; vitamin E, 9,091 mg; vitamin K, 454.5 mg; vitamin B_12_, 3.64 mg; riboflavin, 909.1 mg; d-pantothenic acid, 2,500 mg as calcium pantothenate; niacin, 3,409 mg; and choline, 29,523 mg

^c^The trace mineral premix provided the following quantities of minerals per kg of complete diet: Fe, 257 mg as ferrous sulfate; Zn, 286 mg as zinc sulfate; Mn, 5,710 mg as manganous oxide; Cu, 2,290 mg as copper sulfate; I, 100 mg as calcium iodate; and Se, 85.7 mg as sodium selenite

^d^Pulmotil® 18, tilmicosin phosphate. Elanco Animal Health, Indianapolis, IN, USA

**Table 2:** Analyzed nutrient composition of experimental diets and spray dried plasma (as-fed basis)

Item	Lactation period			Nursery period			Spray dried plasma
	Basal		Creep feed	Phase 1		Phase 2	
Spray dried plasma, %:	‒	0.50	4.75	‒	6.00	‒	
Dry matter, %	88.15	87.93	89.39	88.43	88.16	88.60	89.53
Crude protein, %	16.72	16.87	20.37	22.84	22.34	19.47	80.91
Ash, %	5.47	5.42	6.73	6.02	5.74	5.56	6.92
Acid hydrolyzed ether extract, %	4.78	5.65	3.40	4.16	4.31	3.96	2.39
Gross energy, kcal/kg	3,956	3,972	3,886	3,939	3,976	3,957	4,710
Insoluble dietary fiber, %	17.20	17.90	9.10	9.80	9.50	10.40	1.50
Soluble dietary fiber, %	1.30	1.20	0.40	1.20	0.80	0.90	0.50
Total dietary fiber, %	18.50	19.30	9.50	11.00	10.30	11.30	2.00
Starch, %	26.09	27.12	29.25	26.76	28.28	31.38	‒
Ca, %	0.79	0.72	1.44	1.20	1.17	1.23	0.13
P, %	0.70	0.65	0.97	1.09	1.14	1.05	1.52
Indispensable amino acids, %							
Arg	1.06	1.08	1.09	1.45	1.32	1.18	4.45
His	0.44	0.45	0.49	0.58	0.58	0.49	2.35
Ile	0.73	0.74	0.86	1.04	0.93	0.90	2.41
Leu	1.40	1.45	1.69	1.91	1.93	1.64	7.18
Lys	1.04	1.04	1.55	1.61	1.65	1.42	7.05
Met	0.26	0.25	0.39	0.43	0.46	0.42	0.93
Phe	0.82	0.85	0.90	1.11	1.07	0.92	4.08
Thr	0.65	0.67	0.98	0.99	1.07	0.83	4.89
Trp	0.17	0.20	0.28	0.30	0.33	0.26	1.57
Val	0.81	0.84	1.05	1.11	1.16	0.97	5.50
Total	7.38	7.57	9.28	10.53	10.50	9.03	40.41
Dispensable amino acids, %							
Ala	0.82	0.85	0.94	1.09	1.08	0.93	3.75
Asp	1.70	1.74	1.95	2.41	2.28	1.99	7.75
Cys	0.27	0.28	0.38	0.36	0.46	0.31	2.66
Glu	2.90	2.99	3.22	4.06	3.74	3.41	10.65
Gly	0.73	0.75	0.71	0.89	0.83	0.75	2.68
Pro	0.96	1.00	1.09	1.30	1.26	1.11	4.04
Ser	0.73	0.78	0.82	1.01	1.06	0.78	4.41
Tyr	0.56	0.58	0.64	0.76	0.79	0.64	3.88
Total	8.67	8.97	9.75	11.88	11.50	9.92	39.82
Total amino acids, %	16.05	16.54	19.03	22.41	22.00	18.95	80.23

### Feeding and sample collection

Sows were moved from the gestation facility to individual farrowing crates (2.1 m × 1.5 m) in the farrowing unit on d 107 of gestation. All sows were subjected to heat stress with the temperature in the lactation facility at 26.6 ± 4.8 °C and humidity was 65.9% ± 13.9%. From d 107 of gestation to farrowing, all sows were fed their assigned diet at 2.5 kg/d, which was provided in 2 equal meals, but prior to each morning feeding, feed left from the previous day was removed and the weight was recorded. After farrowing, sows were fed their assigned diet on an ad libitum basis and feed was added to feeders twice daily (at 0615 and 1400 h) until pigs were weaned at 20.9 ± 0.3 d. Orts were collected and weight was recorded on d 10 of lactation and on the day of weaning to calculate average daily feed intake (ADFI) from d 1 to 10, d 10 to weaning, and for the overall lactation period. Sows were weighed at the beginning of the experiment, on d 1 and d 10 of lactation, and on the day of weaning to calculate average daily gain (ADG).

Respiration rates (breaths/min) were measured for all sows by measuring the number of flank movements per minute. Measurements were collected every 2 d (between 0900 and 1000 h) from when sows moved into the lactation facility and until weaning. Sows were induced to farrow on d 114 of gestation and data for litter performance were recorded. On d 1 of lactation, the number of total born pigs, pigs born alive, stillborn pigs, and mummified pigs was recorded from each sow. Pigs underwent routine processing, including umbilical cord and needle teeth clipping, tail docking, castration of male pigs, administration of iron dextran (Uniferon 200, Pharmacosmos A/S, Holbaek, Denmark) and ceftiofur antibiotic (EXCEDE®, Zoetis, Parsippany, NJ, USA), and ear notching within 24 h after birth. Cross-fostering was completed immediately after processing and only within treatment groups. Following normal farm procedures, pigs weighing less than 0.8 kg at birth were euthanized. Weight of pigs that died during the lactation period as well as the reason for death (i.e., crushed by sow, low vitality/starved, or rupture) were recorded. All pigs were weighed on d 10 of lactation and the day prior to weaning. All litters were offered a standard creep feed with 4.75% SDP from d 13 post-farrowing, according to normal farm procedures. Creep feed disappearance was measured by recording the amount provided each day and the amount left in the feeder on the day of weaning.

At weaning, pigs remained with their litter mates and 8 or 10 pigs from each sow, depending on the number of pigs weaned, were randomly selected, and moved to the nursery facility. Pigs were then allotted to the phase 1 diet without or with 6% SDP and housed in mixed sex pens in groups of 4 or 5 pigs per pen, and sex was balanced within treatments. Pigs were fed the phase 1 diets for 14 d post-weaning and all pigs were supplemented with Gentamicin Sulfate (Bimeda Inc., Le Sueur, MN, USA) via the water supply for 3 d starting on d 7 post-weaning due to an outbreak of rotavirus in the facilities. Pigs were then fed the common phase 2 diet without SDP for an additional 22 d. All pigs were allowed ad libitum access to feed and water. Diarrhea scores were assessed visually every other day for 36 d by 2 independent observers using a score from 1 to 5 (1 = normal feces; 2 = moist feces; 3 = mild diarrhea; 4 = severe diarrhea; and 5 = watery diarrhea). Diarrhea frequency was calculated by totaling the number of pen days with diarrhea scores ≥ 3 divided by the total number of pen days multiplied by 100. Individual pig weight was recorded at the beginning of the nursery period and on d 7, 14, and 36. Daily feed allotments were recorded, and feed left in the feeders was weighed on d 7, 14, and 36.

### Blood sampling and chemical analysis

Three blood samples were collected from the jugular vein of each sow on d 1 and 10 after farrowing, and at weaning. Two of the 3 blood samples were collected in vacutainers with ethylenediaminetetraacetic acid (EDTA). These samples were stored on ice immediately after collection and 1 of the EDTA vacutainers was sent to the University of Illinois Veterinary Diagnostic Laboratory for counts of white blood cells, neutrophils, and lymphocytes. The second EDTA vacutainer was centrifuged at 4,000 × *g* for 13 min to recover the plasma and stored at −20 °C until analyzed for malondialdehyde (MDA) and glutathione peroxidase 1 (GPX1) using ELISA kits according to the recommendations from the manufacturer (MyBioSource, Inc., San Diego, CA, USA). The third blood sample was collected in vacutainers without EDTA and blood serum was obtained from this sample by centrifugation at 1,500 × *g* at 4 °C for 15 min. Serum samples were stored at −20 °C until analysis for the following cytokines: interferon-gamma (IFN-γ), interleukin- (IL-)1α, IL-1β, IL-1 receptor antagonist, IL-2, IL-4, IL-6, IL-8, IL-10, IL-12, IL-18, and tumor necrosis factor-α (TNF-α). Cytokines were analyzed using a MILLIPLEX kit (EMD Millipore Corporation, Billercia, MA, USA) in a MagPix instrument with ProcartaPlex multiplex technology (R&D Systems, Inc., Minneapolis, MN, USA).

All diets and the SDP ingredient were analyzed in duplicate for concentrations of gross energy using an isoperibol bomb calorimeter (Model 6400, Parr Instruments, Moline, IL, USA), and for nitrogen by combustion (method 990.03; [9]) using a LECO FP628 analyzer (LECO Corp., Saint Joseph, MI, USA). Crude protein was calculated as nitrogen × 6.25. Dry matter was analyzed in all diets and in SDP by oven drying at 135 °C for 2 h (method 930.15; [9]) and dry ash was analyzed as well (method 942.05; [9]). Concentrations of Ca and P in diets and SDP were analyzed using inductively coupled plasma-optical emission spectrometry (ICP-OES; Avio 200, PerkinElmer, Waltham, MA, USA). Sample preparation included dry ashing at 600 °C for 4 h (method 985.01 A–C; [9]) and wet digestion with nitric acid (method 3050 B; [10]). All diets and SDP were also analyzed for insoluble- and soluble-dietary fiber (method 991.43; [9]) using the Ankom Dietary Fiber Analyzer (Ankom Technology, Macedon, NY, USA), and total dietary fiber was calculated as the sum of insoluble- and soluble-dietary fiber. Acid hydrolyzed ether extract was analyzed using the acid hydrolysis filter bag technique (Ankom^HCl^ Hydrolysis System; Ankom Technology, Macedon, NY, USA) followed by crude fat extraction using petroleum ether (Ankom^XT15^ Extractor; Ankom Technology, Macedon, NY, USA). At the Agricultural Experiment Station Chemical Laboratories at the University of Missouri (Columbia, MO, USA), all diets and the SDP ingredient were analyzed for amino acids on a Hitachi Amino Acid Analyzer, Model No. L8800 (Hitachi High Technologies America, Inc., Pleasanton, CA, USA) using ninhydrin for postcolumn derivatization and norleucine as the internal standard (method 982.30 E (a–c); [9]), and all diets were analyzed for total starch using the glucoamylase procedure (method 979.10; [9]).

### Calculations and statistical analysis

At the conclusion of the experiment, data for sow body weight (BW) loss, ADFI, and pig mortality during lactation (calculated as the percentage of live born pigs that died before weaning after adjusting for cross-fostering) were calculated. Total live litter birth weight, live litter birth weight after cross fostering, litter weight on d 10 of lactation and at weaning, and litter ADG were calculated as well. Average pig weight at birth, at d 10 of lactation, and at weaning was calculated, as well as pig ADG during lactation and creep feed disappearance. During the nursery period, data collected for pig weight and feed allowance were summarized and ADG, ADFI, and gain to feed ratio (G:F) were calculated for each pen and treatment group.

Normality of residuals were verified, and outliers were identified using the UNIVARIATE and BOXPLOT procedures of SAS, respectively (SAS Inst. Inc., Cary, NC, USA). Outliers were removed if the value deviated from the 1^st^ or 3^rd^ quartiles by more than 3 times the interquartile range [11]. Data were analyzed using a split-plot design with sow treatment (diet within parity) as the main plot and nursery diet as the sub-plot. For the sow portion of the experiment, data were analyzed as a 2 × 2 factorial arrangement of treatments using SAS PROC GLIMMIX with binomial distribution for data related to pig mortality or Poisson distribution for data related to the number of pigs per litter. The PROC MIXED was used for all other data analyses. The sow was the experimental unit for all analyses and date of breeding was the blocking factor. For both PROC GLIMMIX and PROC MIXED, the statistical model included the fixed effects of diet, parity, and parity by diet interaction and the random effect of block. Blood samples were collected from the same sows during the experiment, therefore, data for blood analyses were analyzed as repeated measures using the PROC MIXED and REPEATED procedures of SAS. The model included diet, parity group, day, and all 2- and 3-way interactions as main effects, day as the time effect, and sow as the subject. However, the interactions between diet and day, parity and day, and diet, parity, and day were not significant, therefore, contrast statements were used with coefficients for equally spaced treatments to determine linear and quadratic effects of day on blood variables. For the nursery part of the experiment, wean group was the blocking factor and pen was the experimental unit for all analyses. The model included the fixed effect of sow treatment (main plot), nursery diet (sub-plot), and the interaction between sow treatment and nursery diet and the random effect of block and block by sow treatment.

Treatment means were estimated for each mortality-related variable using the LSMEANS statement with the inverse link option in PROC GLIMMIX. Data for plasma MDA and GPX1 and serum cytokines were transformed using base-10 log prior to analysis in PROC MIXED to obtain a normal distribution. Treatment means were reported for all other variables using the LSMEANS statement in PROC MIXED, and if an interaction was significant, means were separated using the PDIFF option. Statistical significance and tendencies were considered at *P* < 0.05 and 0.05 ≤ *P* < 0.10, respectively.

## Results

Four sows were removed from the experiment: 2 young sows fed the diet with 0.5% SDP were removed before farrowing; and 1 mature sow fed the diet with 0.5% SDP and 1 young sow fed the diet without SDP were removed within 2 d after farrowing.

### Lactation

**Fig. 1:**
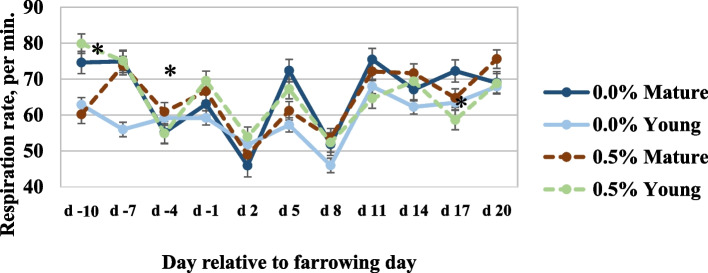
Effects of dietary spray dried plasma on respiration rate of young or mature sows housed at 29.0 °C on sow respiration rates (number of respirations per minute). ^*^P < 0.05 for the interaction between inclusion of SDP and parity group

Respiration rates measured for young sows fed the diet with 0.5% SDP were greater 7 d prior to farrowing compared with young sows fed the diet without SDP (Fig. 1), but respiration rates for mature sows was not influenced by diet (interaction, *P* < 0.05). Respiration rates 5 d after farrowing was not influenced by diet for either sow parity group, but mature sows fed the diet without SDP had greater respirations per minute than young sows fed the diet without SDP, however if SDP was included in the diet, no difference between young and mature sows was observed (interaction, *P* < 0.05).

The parity of young sows was less (*P* < 0.05) than the parity of mature sows (Table 3), but within the 2 parity groups, parity did not differ between dietary treatments. Body weight of sows was not affected by dietary treatment at the initiation of the experiment, at farrowing, or at weaning. However, mature sows had greater (*P* < 0.05) BW throughout the experiment compared with young sows. From d 10 to 20 of lactation, young sows fed the diet with 0.5% SDP had less BW loss compared with young sows fed the diet without SDP, but BW loss for mature sows was not affected by diet (interaction, *P* < 0.05). During the entire lactation period (d 1 to 20), sows fed the diet with 0.5% SDP tended to have less (*P* < 0.10) BW loss than sows fed the diet without SDP, regardless of parity. Mature sows had less (*P* < 0.05) BW loss than young sows from d 1 to 10 and from d 1 to 20 of lactation. The ADFI of sows was not affected by diet, but the ADFI of mature sows was greater (*P* < 0.05) than the ADFI of young sows. There was no feed refusals before farrowing regardless of the diet being fed or parity of sows.

**Table 3:** Reprodcutive performance of young and mature sows during lactation^1^

Parity:	Young		Mature		Pooled SEM	*P*-value^2^		
Spray dried plasma, %:	‒	0.50	‒	0.50		D	P	D × P
Parity	1.52	1.48	4.35	4.44	0.20	0.928	< 0.001	0.746
Body weight, kg								
d 107 gestation	227	224	254	246	3.93	0.142	< 0.001	0.632
d 1 lactation	214	210	240	235	4.30	0.274	< 0.001	0.930
d 10 lactation	205	202	234	234	5.84	0.648	< 0.001	0.712
d 20 lactation	193	194	226	222	4.61	0.740	< 0.001	0.706
Average daily gain, kg								
d 1 to 10 lactation	−0.92	−0.86	−0.60	−0.13	0.27	0.114	0.002	0.236
d 10 to 20 lactation	−1.37^b^	−0.78^a^	−0.98^ab^	−1.05^ab^	0.32	0.031	0.624	0.006
d 1 to 20 lactation	−1.07	−0.83	−0.72	−0.58	0.12	0.065	0.005	0.612
Average daily feed intake, kg								
d 1 to 10 lactation	3.92	4.14	4.50	4.63	0.28	0.301	0.003	0.790
d 10 to 20 lactation	5.45	5.23	6.05	6.13	0.17	0.697	< 0.001	0.380
d 1 to 20 lactation	4.68	4.69	5.28	5.43	0.18	0.590	< 0.001	0.649

^a,b^Means within a row lacking a common superscript letter differ (*P* < 0.05)

^1^Data are least square means of 15 to 21 observations per treatment

^2^*P*-values were calculated for the main effects of diet (D) and parity (P) and the interaction between diet and parity (D × P)

There was no effect of diet on number of total pigs born or pigs born alive (Table 4), but there was a greater (*P* < 0.05) number of total pigs born from mature sows compared with young sows. There was a tendency for a reduced number of still born pigs from young sows fed the diet without SDP than with SDP, but the number of still born pigs from mature sows did not differ between treatments (interaction, *P* < 0.10). There was a tendency for fewer (*P* < 0.10) mummified pigs born from sows fed the diet with 0.5% SDP compared with sows fed the diet without SDP, regardless of parity. No differences between treatments were observed for total litter weight or individual pig weight at birth, but pigs born from sows fed the diet without SDP tended to have greater (*P* < 0.10) BW at weaning and had greater (*P* < 0.05) ADG during lactation compared with pigs born from sows fed the diet with 0.5% SDP. Creep feed disappearance during lactation was greater (*P* < 0.05) for pigs from mature sows fed the diet without SDP compared with pigs from sows fed the diet with 0.5% SDP, but creep feed disappearance did not differ between treatments for young sows (interaction, *P* < 0.05). Dietary treatment did not affect the proportion of pigs from mature sows that died before weaning, but young sows fed the diet with 0.5% SDP tended to have reduced pig mortality compared with mature sows (interaction, *P* < 0.10). There was a tendency (*P* < 0.10) for increased percentage of pigs crushed by mature sows than young sows, and there was a greater (*P* < 0.05) percent of low vitality or starved pigs from mature sows than young sows, but the percent of low vitality or starved pigs was less (*P* < 0.05) from sows fed the diet with 0.5% SDP than from sows fed the diet without SDP, regardless of parity group.

**Table 4:** Productive performance of litters from young and mature sows during lactation^1^

Parity:	Young		Mature		Pooled SEM	*P*-value^2^		
Spray dried plasma, %:	‒	0.50	‒	0.50		D	P	D × P
Pigs per litter, n								
Total born	14.52	14.62	17.56	16.33	0.95	0.581	0.014	0.509
Born alive	14.00	13.86	15.88	14.56	0.90	0.434	0.158	0.538
After cross-fostering	14.33	14.05	15.00	15.00	0.89	0.869	0.365	0.869
Still born	0.31^z^	0.65^yz^	1.14^y^	0.81^yz^	0.26	0.504	0.011	0.071
Mummified	0.19	0.05	0.47	0.13	0.11	0.052	0.177	0.965
At d 10 of lactation	12.43	12.96	12.76	12.81	0.83	0.818	0.815	0.862
At weaning	12.24	12.85	12.24	12.44	0.83	0.625	0.805	0.808
Litter weight, kg								
Live at birth	19.11	19.63	21.52	19.93	0.90	0.506	0.095	0.199
After cross-fostering	19.72	20.01	20.33	20.70	0.76	0.570	0.262	0.950
At d 10 of lactation	44.14	44.85	42.91	43.51	1.83	0.586	0.285	0.965
At weaning	69.49	70.14	67.90	67.24	3.08	0.998	0.254	0.739
Litter average daily gain, kg	2.48	2.50	2.48	2.33	0.13	0.425	0.306	0.310
Creep feed disappearance^3^, g/d	72.35^ab^	100.98^a^	102.43^a^	69.04^b^	35.46	0.823	0.931	0.007
Individual pig weight, kg								
Live at birth	1.37	1.44	1.42	1.41	0.05	0.608	0.797	0.428
At d 10 of lactation	3.49	3.47	3.51	3.41	0.08	0.454	0.881	0.622
At weaning	5.60	5.49	5.76	5.45	0.13	0.092	0.612	0.421
Pig average daily gain, kg	0.21	0.20	0.22	0.20	0.01	0.037	0.649	0.539
Pig mortality^4^, %								
Died prior to weaning	14.74^yz^	9.10^z^	18.37^y^	19.24^y^	3.30	0.162	0.002	0.088
Crushed by sow	7.35	5.63	8.01	10.90	2.12	0.905	0.080	0.176
Low vitality/starved	6.11	3.00	8.96	6.21	2.17	0.034	0.030	0.513
Rupture	0.33	0.34	0.78	0.83	0.47	0.963	0.312	0.981

^a,b^Means within a row lacking a common superscript letter differ (*P *< 0.05)

^y,z^Means within a row lacking a common superscript letter differ (*P *< 0.10)

^1^Data are least square means of 15 to 21 observations per treatment

^2^*P*-values were calculated for the main effects of diet (D) and parity (P) and the interaction between diet and parity (D × P)

^3^Creep feed was fed from d 13 of lactation until weaning

^4^Mortality was calculated as the percentage of live born pigs that died before weaning after adjusting for cross-fostering

There were no interactions between sow treatment and day for any of the analyzed blood parameters (Table 5). White blood cell counts did not differ between treatments, regardless of sow parity group, but young sows fed the diet with 0.5% SDP had greater white blood cell counts than mature sows fed the diet with 0.5% SDP (interaction, *P* < 0.05). Neutrophils as a percent of white blood cells were greater (*P* < 0.05) in mature sows than young sows, whereas lymphocytes as a percent of white blood cells were less (*P* < 0.05) in mature sows than young sows. No difference was observed for the oxidative stress marker MDA among treatments, whereas GPX1 was less (*P* < 0.05) in mature sows than young sows. All serum cytokines increased (*P* < 0.05) in sows fed the diet with 0.5% SDP compared with sows fed the diet without SDP, and the concentration of all cytokines, except IFN-γ, was greater (*P* < 0.05) in mature sows compared with young sows. The day when blood samples were collected influenced blood parameters, regardless of sow parity or diet. Concentrations of white blood cells, MDA, and GPX1 increased from d 1 to d 10 and from d 10 to d 20 of lactation (quadratic, *P* < 0.05). Serum concentrations of IL-1α, IL-2, IL-4, IL-6, IL-10, IL-12, IL-18, and TNF-α linearly increased (*P* < 0.05) from farrowing to weaning.

**Table 5:** Blood cell counts, indicators of oxidative stress, and cytokines of young and mature sows during lactation^1,2^

Parity:	Young		Mature		Pooled SEM	*P*-value^3^			Day of lactation			Pooled SEM	*P*-value^4^	
SDP, %:	‒	0.50	‒	0.50		D	P	D × P	1	10	20		L	Q
WBC, × 10^3^/µL	12.19^ab^	13.06^a^	11.82^ab^	10.72^b^	0.58	0.799	0.003	0.031	10.29	13.30	12.26	0.50	< 0.001	< 0.001
Neutrophils^5^	51.43	52.51	56.87	56.24	1.72	0.893	0.010	0.622	53.32	53.84	55.62	1.43	0.240	0.716
Lymphocytes^5^	39.18	36.43	31.81	33.80	1.57	0.808	0.002	0.135	36.12	35.74	34.06	1.27	0.230	0.672
Oxidative stress^6^														
MDA, nmol/mL	1.45	1.64	1.49	1.46	0.16	0.282	0.388	0.160	1.26	1.69	1.62	0.16	< 0.001	0.005
GPX1, ng/mL	0.43	0.41	0.35	0.31	0.04	0.453	0.019	0.777	0.32	0.42	0.37	0.03	0.013	< 0.001
Cytokines^6^, ng/mL														
IFN-γ	34.78	67.02	36.84	68.75	15.67	0.002	0.837	0.938	44.51	52.12	51.62	11.32	0.003	0.053
IL-1α	0.33	0.75	0.69	1.22	0.16	< 0.001	< 0.001	0.506	0.62	0.69	0.72	0.08	< 0.001	0.499
IL-1β	1.64	4.77	3.88	8.12	1.29	< 0.001	0.002	0.465	3.83	3.99	4.07	0.55	0.110	0.729
IL-1Ra	1.86	4.23	3.90	7.22	1.03	< 0.001	< 0.001	0.552	4.63	3.35	3.70	0.62	0.002	< 0.001
IL-2	1.78	4.63	3.80	7.23	1.03	< 0.001	0.002	0.421	3.55	4.00	4.12	0.49	< 0.001	0.198
IL-4	9.60	28.93	27.46	62.19	10.77	< 0.001	< 0.001	0.583	22.73	27.59	28.81	4.23	< 0.001	0.105
IL-6	0.97	2.14	1.90	4.35	0.58	< 0.001	0.001	0.918	1.90	2.05	2.17	0.21	0.001	0.800
IL-8	0.17	0.23	0.20	0.47	0.06	0.003	0.031	0.173	0.42	0.18	0.19	0.03	< 0.001	< 0.001
IL-10	4.37	9.93	8.46	17.73	2.30	< 0.001	0.002	0.835	8.53	8.91	9.53	0.90	0.015	0.755
IL-12	1.76	3.28	2.91	5.51	0.66	< 0.001	0.002	0.961	2.91	3.12	3.27	0.35	< 0.001	0.661
IL-18	7.25	17.46	15.11	32.21	4.49	< 0.001	0.001	0.762	14.42	16.08	16.86	1.97	< 0.001	0.354
TNF-α	0.13	0.37	0.24	0.69	0.13	< 0.001	0.041	0.985	0.28	0.30	0.31	0.05	0.050	0.907

^a,b^Means within a row lacking a common superscript letter differ (*P* < 0.05)

^1^Data are least square means of 13 to 21 observations per treatment

^2^*GPX1* Glutathione peroxidase 1, *IFN-γ* Interferon-gamma, *IL*- Interleukin-, *IL-1Ra* Interleukin-1 receptor antagonist, *MDA* Malondialdehyde, *SDP* Spray dried plasma, *TNF-α* Tumor necrosis factor-α, *WBC* White blood cell

^3^*P*-values were calculated for the main effects of diet (D) and parity (P) and the interaction between diet and parity (D × P)

^4^*P*-values were calculated to test the linear (L) and quadratic (Q) effects of day

^5^Neutrophils and lymphocytes are a % of white blood cells measured in the whole blood

^6^Values were log10 transformed before analysis to obtain a normal distribution, but data are shown as back-transformed least square means

### Nursery

Pigs weaned from mature sows fed the diet without SDP had greater ADG from d 7 to 14 post-weaning if they were fed the SDP diet than the diet without SDP (Table 6), but the ADG of pigs weaned from the other sow groups was not influenced by post-weaning diet (interaction, *P* < 0.10). In phase 1, pigs weaned from young sows fed the diet without SDP or from mature sows fed the diet without or with 0.5% SDP had greater G:F when fed the diet with 6% SDP compared with pigs fed the diet without SDP, but the G:F of pigs weaned from young sows fed the diet with 0.5% SDP was not affected by phase 1 dietary treatment (interaction, *P* < 0.05). In phase 2, pigs weaned from young sows fed the diet with 0.5% SDP had reduced G:F if fed a phase 1 diet with SDP compared with pigs fed a phase 1 diet without SDP, whereas phase 1 diet did not affect G:F of pigs weaned from the other sow treatments (interaction, *P* < 0.05). Regardless of sow treatment, pigs fed the phase 1 diet with 6% SDP had greater (*P* < 0.05) ADG, ADFI, and BW at the end of phase 1 and greater (*P* < 0.05) ADFI and final BW at the end of phase 2 than pigs fed the phase 1 diet without SDP. During the overall nursery period, pigs fed the phase 1 diet with 6% SDP had greater (*P* < 0.05) ADG and ADFI than pigs fed the phase 1 diet without SDP, but the overall G:F was not affected by phase 1 diet.

**Table 6:** Influence of sow treatment group and phase 1 diet on growth performance of weaned pigs^1–3^

Parity:	Young				Mature				Pooled SEM	*P*-value^4^		
Sow SDP, %:	‒		0.50		‒		0.50					
Nursery SDP, %:	‒	6.00	‒	6.00	‒	6.00	‒	6.00		S	N	S × N
d 1 to 7												
Initial BW, kg	5.74	5.70	5.45	5.51	5.69	5.85	5.70	5.61	0.17	0.441	0.808	0.851
ADG, g	35	109	37	111	8	101	25	95	11.09	0.377	< 0.001	0.557
ADFI, g	105	157	111	163	90	155	105	147	9.88	0.610	< 0.001	0.618
G:F	0.27	0.62	0.34	0.68	0.19	0.66	0.12	0.56	0.07	0.233	< 0.001	0.646
d 7 BW, kg	5.91	6.48	5.67	6.23	5.77	6.53	5.85	6.27	0.20	0.700	< 0.001	0.799
d 7 to 14												
ADG, g	171^yz^	188^yz^	188^yz^	195^yz^	129^z^	202^y^	181^yz^	210^y^	19.26	0.314	< 0.001	0.051
ADFI, g	231	236	240	252	205	253	243	271	17.22	0.239	0.004	0.209
G:F	0.70^ab^	0.76^ab^	0.81^a^	0.81^a^	0.62^b^	0.82^a^	0.74^ab^	0.78^ab^	0.04	0.216	< 0.001	0.008
d 14 BW, kg	7.10	7.82	6.98	7.67	6.69	7.84	6.98	7.59	0.26	0.911	< 0.001	0.597
d 1 to 14												
ADG, g	104	148	112	150	70	146	98	154	12.75	0.344	< 0.001	0.115
ADFI, g	169	196	173	206	147	199	169	210	11.81	0.460	< 0.001	0.477
G:F	0.58^bcd^	0.74^a^	0.63^abc^	0.72^ab^	0.44^d^	0.71^ab^	0.52^ cd^	0.72^ab^	0.04	0.206	< 0.001	0.021
d 14 to 36												
ADG, g	488	511	499	477	494	520	504	509	16.82	0.693	0.341	0.151
ADFI, g	693	744	708	727	710	784	721	755	24.88	0.648	< 0.001	0.445
G:F	0.71^ab^	0.69^abc^	0.71^a^	0.66^c^	0.70^abc^	0.67^bc^	0.70^ab^	0.68^abc^	0.01	0.354	< 0.001	0.026
Final BW, kg	17.95	18.76	17.96	18.18	17.64	19.38	18.13	18.86	0.54	0.895	0.006	0.383
Overall												
ADG, g	341	363	349	352	332	374	344	367	12.53	0.987	0.002	0.246
ADFI, g	481	517	498	517	488	548	503	541	17.54	0.666	< 0.001	0.530
G:F	0.69	0.70	0.70	0.68	0.69	0.69	0.69	0.69	0.01	0.905	0.506	0.204

^a–d^Means within a row lacking a common superscript letter differ (*P* < 0.05)

^y,z^Means within a row lacking a common superscript letter differ (*P* < 0.10)

^1^Data are least square means of 13 to 19 observations per diet

^2^*ADFI* Average daily feed intake, *ADG* Average daily gain, *BW* Body weight, *G:F* Gain to feed ratio, *SDP* Spray dried plasma

^3^All pigs were fed a diet without or with 6% SDP for 14 d post-weaning, and then fed a common diet with no SDP from d 14 to 36 post-weaning

^4^*P*-values were calculated for the main effects of sow treatment (S) and nursery diet (N) and the interaction between sow treatment and nursery diet (S × N)

Pigs weaned from mature sows had less diarrhea in phase 2 when fed the phase 1 diet with 6% SDP compared with pigs fed the phase 1 diet without SDP (Table 7), but the incidence of diarrhea in phase 2 for pigs weaned from young sows was not affected by phase 1 dietary treatment (interaction, *P* < 0.05). Regardless of sow treatment, pigs fed the phase 1 diet with 6% SDP had reduced (*P* < 0.05) diarrhea in phase 1 and phase 2 compared with pigs fed the phase 1 diet without SDP. The frequency of diarrhea in phase 2 and overall was less (*P* < 0.05) for pigs fed the phase 1 diet with 6% SDP compared with pigs fed the phase 1 diet without SDP.

**Table 7:** Influence of sow treatment group and phase 1 diet on diarrhea score and frequency of diarrhea of weaned pigs^1,2^

Parity:	Young				Mature				Pooled SEM	*P*-value^3^		
Sow SDP, %:	‒		0.50		‒		0.50					
Nursery SDP, %:	‒	6.00	‒	6.00	‒	6.00	‒	6.00		S	N	S × N
Diarrhea score^4^												
d 1 to 6	1.82	1.90	1.95	1.90	2.05	1.87	1.94	2.07	0.09	0.481	0.946	0.195
d 8 to 14	2.93	2.92	3.23	2.93	3.03	2.90	3.06	2.80	0.10	0.351	< 0.001	0.112
d 1 to 14	2.47	2.48	2.69	2.49	2.59	2.49	2.57	2.47	0.08	0.499	0.012	0.215
d 16 to 36	2.36^abc^	2.32^abc^	2.37^abc^	2.34^abc^	2.33^ab^	2.18^c^	2.41^a^	2.21^bc^	0.05	0.359	< 0.001	0.028
d 1 to 36	2.40	2.38	2.49	2.40	2.43	2.30	2.47	2.32	0.05	0.491	< 0.001	0.156
Frequency of diarrhea^5^												
d 1 to 6	8.77	12.28	19.30	5.26	18.75	25.00	20.00	22.22			0.068	
d 8 to 14	59.21	67.11	78.95	65.79	64.06	59.38	71.67	63.33			0.206	
d 1 to 14	37.59	43.61	53.38	39.85	44.64	44.64	49.52	45.71			0.243	
d 16 to 36	17.22	16.75	20.10	18.18	19.89	7.39	21.21	13.94			0.015	
d 1 to 36	25.15	27.19	33.04	26.61	29.51	21.88	32.22	26.30			0.042	

^a–c^Means within a row lacking a common superscript letter differ (*P* < 0.05)

^1^Data are least square means of 13 to 19 observations per diet

^2^*SDP* Spray dried plasma

^3^*P*-values were calculated for the main effects of sow treatment (S) and nursery diet (N) and the interaction between sow treatment and nursery diet (S × N)

^4^Diarrhea scores were visually assessed every other day by 2 independent observers for 36 days. Diarrhea score = 1, normal feces; 2, moist feces; 3, mild diarrhea; 4, severe diarrhea; 5, watery diarrhea

^5^Frequency = (number of pen days with diarrhea scores ≥ 3/pen days) × 100

## Discussion

Pigs lack functional sweat glands and therefore are inefficient in thermoregulation and highly susceptible to heat stress [12]. Heat stress was estimated to cost the United States swine industry $299 million per year [13], with loss in sow productivity alone estimated at $113 million per year [13]. The temperature used in the current experiment to stimulate heat stress is in agreement with published data, where sows used in heat stress experiments were housed at temperatures between 27 to 32 °C [14]. Physiological responses to heat stress include increases in respiration rate and body temperature with respiration rate being a more sensitive indicator of heat stress than body temperature [1]. Sows housed in a thermoneutral environment generally had a respiration rate of approximately 30 breaths per minute, whereas the respiration rate of heat stressed sows ranged between 50 and 80 breaths per minute [1]. Respiration rates measured in the current experiment are in agreement with previous values [1].

Heat stress is also characterized by decreased feed intake of sows [1], resulting in greater weight loss during lactation and reduced litter performance [14]. Sows of parity 1 and 2 fed a diet with 0.25% or 0.50% SDP had increased feed intake during summer months compared with sows fed a diet without SDP [4], but data from the current experiment agree with Carter et al. [5] who reported that feed intake of parity 1 to 3 sows was not affected by dietary SDP. During lactation, sows often need to mobilize body reserves to support milk production leading to increased weight loss of the sow [15]. Results from the current experiment indicating that weight loss tended to be reduced during lactation of sows fed dietary SDP agrees with previous data [15]. Because there was no impact of SDP on ADFI of sows, it is speculated that addition of SDP to the diets resulted in improved digestibility or utilization of energy or nutrients in the diets. Inclusion of SDP in diets for weanling pigs increases digestibility and absorption of nutrients [16, 17], but this has not been demonstrated for sows.

Crenshaw et al. [6] reported decreased still born pigs from sows fed 0.5% or 2.5% SDP. Litter size, pig birth weight, sow BW, and sow parity can impact the number of still born pigs [6], and as sow parity increases, the probability of still born pigs increases [18], which was also observed in the current experiment. The observation that ADG of pigs during lactation decreased when sows were fed 0.5% dietary SDP is a result of the numerical increase in the number of pigs weaned from sows fed SDP, because litter daily gain was not influenced by SDP. This is in contrast with results indicating an increase in growth rate of pigs during lactation from sows fed a diet supplemented with 1% SDP [15]. It therefore appears that the 0.5% inclusion of SDP in sow diets was not sufficient to increase pig growth rate. Vitality of pigs from sows fed dietary SDP in the current experiment was increased, and Kim et al. [15] indicated that milk production increased, and thus milk consumption by pigs increased if sows were fed a diet containing SDP. However, milk production from sows was not measured in either experiment. Overall, our data are in agreement with Frugé et al. [19] and Carter et al. [5] indicating limited effects of dietary SDP on litter performance, but further research is needed to elucidate the mechanisms of improved pig vitality during lactation from sows fed dietary SDP.

Spray dried plasma contains immunoglobulins that are hypothesized to have immunomodulatory effects on pigs, which is more important during periods of increased stress than in periods without stress [20]. Including 1% SDP in diets fed to sows during late gestation and throughout lactation reduced serum TNF-α 7 d after farrowing [15], although data from Crenshaw et al. [6] indicated no influence of 0.5% or 2.5% dietary SDP on serum cytokines in sows 2 d before or 4 d after farrowing. Thus, results from the current experiment indicating that both pro- and anti-inflammatory serum cytokines increased for sows fed 0.5% dietary SDP are in contrast with previous data [6]. Age is a significant factor affecting cytokine production, and serum cytokines were greater for mature sows than young sows, which has also been previously reported [21], but pigs have high individual variation in cytokine production [21]. Cytokines are secreted by innate immune cells, such as white blood cells, in response to various stimuli related to inflammation and infection in the animal [22], but this is unlikely to have influenced concentrations of cytokines because white blood cells were not affected by dietary SDP.

There is limited information about blood cytokines in sows fed SDP. Previous data indicate a general reduction in proinflammatory cytokines and an increase in anti-inflammatory cytokines for sows under normal ambient temperature suggesting that dietary SDP favorably modulated immune/cytokine response [23]. Similar modulations of cytokine results were observed in uterine tissue of pregnant mice exposed to transport stress or lipopolysaccharides [24, 25]. Heat stress is generally associated with more gut leakage, higher endotoxin uptake, and immune system stimulation. The greater cytokine profile in the heat stressed sows used in the present experiment indicate that under heat stress conditions both pro and anti-inflammatory cytokine are elevated, which may be due to a more robust immune response of sows fed SDP vs. control sows because they need to combat gut leakage associated endotoxin uptake during heat stress. It is also possible that the cytokines interacted with adapted heat shock protein effects because of the heat stress. Further research is needed to better explain this response and should be compared in a study evaluating cytokine profile response in lactating sows fed diets without or with SDP under ambient or heat stress conditions. In addition, increased cytokine production activates the immune system, which increases nutrient requirements to maintain immune cell synthesis and leaving less nutrients available for growth of the animal [26, 27], but sow performance in the current experiment was not reduced due to dietary SDP, and there was, therefore, no negative impact of the increased concentration of cytokines on sow performance.

Spray dried plasma is often included in diets for weanling pigs and at greater concentrations in the diet compared with sow diets [20]. Including SDP in diets fed to weanling pigs improves growth performance of pigs and reduces diarrhea incidence [28, 29], and data from the current experiment agree with these observations. Dietary SDP is usually included in diets for weanling pigs during the initial 1 to 2 week post-weaning, because improvements in growth performance are not significant thereafter [30]. Thus, the increased growth performance in phase 1 and the increased ADG for the overall experimental period agrees with previous data. The observation that final BW at the end of phase 2 was greater for pigs fed the phase 1 diet with SDP compared with pigs fed the phase 1 diet without SDP demonstrates that the benefits of feeding SDP in phase 1 were maintained in phase 2.

The effect of supplementing sow diets with SDP on the subsequent performance of their offspring post-weaning has only recently been evaluated [15]. Pigs fed diets without SDP and weaned from sows fed 1% SDP had greater ADG than pigs weaned from sows fed a diet without SDP [15]. The observation that ADG did not differ between pigs fed a diet without SDP and with SDP when weaned from sows fed SDP, indicates that inclusion of SDP in lactation diets improved performance of pigs after weaning, which agrees with previous data [15]. The reason for these changes in weanling pig performance may be that sows fed a diet containing SDP during lactation may secrete more immune cells in milk, and thereby prepare pigs for the stress of weaning. However, research to address this hypothesis needs to be conducted.

Diarrhea caused by bacterial and viral pathogens is a common problem post-weaning leading to economic losses due to pig mortality, morbidity, and decreased efficiency [30]. Inclusion of SDP in post-weaning diets has been reported to reduce diarrhea incidence and improve intestinal function of weanling pigs [20, 31], and the observation that, regardless of SDP inclusion in the sow diet, pigs fed SDP post-weaning had reduced diarrhea incidence is in agreement with previous data [20]. Dietary SDP is also more effective in improving intestinal function and performance of pigs when housed in an environment undergoing a sanitation challenge [31]. The reduction in diarrhea incidence observed for pigs fed SDP was carried over into the subsequent phase when dietary SDP was not included in the diet indicating that the phase 1 diet with SDP maintained the function of the intestinal barrier of pigs infected with rotavirus, which agrees with Corl et al. [32]. Increasing feed intake and limiting weight loss of the sow during lactation can result in improvements in milk yield, which benefits litter performance [33]. Including probiotics in sow diets modify the intestinal ecosystem of pigs resulting in less diarrhea and improvements in intestinal barrier function post-weaning [34]. However, data on the effect of SDP fed to sows on growth performance of their offspring after weaning have not been reported. Thus, the observation that diarrhea incidence did not differ between pigs fed a diet without SDP and with SDP when weaned from young sows fed SDP, indicates that inclusion of SDP in lactation diets may have improved the intestinal barrier function of pigs after weaning.

## Conclusions

Feeding 0.5% dietary SDP to sows may reduce the number of mummified pigs born and increase pig vitality during lactation, but this does not appear to be a result of an increase in systemic cytokines of sows fed 0.5% dietary SDP. Further research is needed to assess if the benefits outweigh the potential risks of inflammatory responses, particularly when considering the overall health and productivity of both sows and their offspring during gestation and lactation. In addition, feeding a diet with 0.5% SDP to sows during lactation did not improve post-weaning growth performance of pigs, but 6% dietary SDP in the phase 1 nursery diet improved growth performance parameters and decreased diarrhea incidence of pigs compared with pigs fed a diet without SDP.

## Data Availability

The datasets used and/or analyzed during the current study are available from the corresponding author upon reasonable request.

## Abbreviations

ADFI: Average daily feed intake
ADG: Average daily gain
BW: Body weight
EDTA: Ethylenediaminetetraacetic acid
G:F: Gain to feed ratio
GPX1: Glutathione peroxidase 1
IL-: Interleukin-
IFN-γ: Interferon-gamma
MDA: Malondialdehyde
SDP: Spray dried plasma
TNF-α: Tumor necrosis factor-α
